# Ionome mapping and amino acid metabolome profiling of *Phaseolus vulgaris* L. seeds imbibed with computationally informed phytoengineered copper sulphide nanoparticles

**DOI:** 10.1186/s11671-023-03953-y

**Published:** 2024-01-04

**Authors:** Nandipha L. Botha, Karen J. Cloete, Žiga Šmit, Kristina Isaković, Mahmood Akbari, Razieh Morad, Itani Madiba, Oladipupo Moyinoluwa David, Luis P. M. Santos, Admire Dube, Primoz Pelicon, Malik Maaza

**Affiliations:** 1 UNESCO-UNISA Africa Chair in Nanosciences and Nanotechnology Laboratories, College of Graduate Studies, University of South Africa, Muckleneuk Ridge, PO Box 392, Pretoria, 0003 South Africa; 2 Nanosciences African Network (NANOAFNET), iThemba LABS-National Research Foundation, PO Box 722, Somerset West, Western Cape Province 7129 South Africa; 3 Faculty of Mathematics and Physics, University of Ljubljana, Jadranska 19, 1000 Ljubljana, Slovenia; 4 Jožef Stefan Institute, Jamova 39, 1001 Ljubljana, Slovenia; 5 School of Pharmacy, University of the Western Cape, Bellville, 7535 South Africa; 6 Graduate Program in Materials Science and Engineering, Federal University of Ceará, Campus of PICI, Fortaleza, CE 60440-900 Brazil

**Keywords:** Amino acid, Avocado-seed-extract, CuS, Nanofertilizer, *Phaseolus vulgaris*

## Abstract

This study reports the effects of a computationally informed and avocado-seed mediated Phyto engineered CuS nanoparticles as fertilizing agent on the ionome and amino acid metabolome of Pinto bean seeds using both bench top and ion beam analytical techniques. Physico-chemical analysis of the Phyto engineered nanoparticles with scanning-electron microscopy, transmission electron microscopy, X-ray diffraction, and Fourier Transform Infrared Spectroscopy confirmed the presence of CuS nanoparticles. Molecular dynamics simulations to investigate the interaction of some active phytocompounds in avocado seeds that act as reducing agents with the nano-digenite further showed that 4-hydroxybenzoic acid had a higher affinity for interacting with the nanoparticle’s surface than other active compounds. Seeds treated with the digenite nanoparticles exhibited a unique ionome distribution pattern as determined with external beam proton-induced X-ray emission, with hotspots of Cu and S appearing in the hilum and micropyle area that indicated a possible uptake mechanism via the seed coat. The nano-digenite also triggered a plant stress response by slightly altering seed amino acid metabolism. Ultimately, the nano-digenite may have important implications as a seed protective or nutritive agent as advised by its unique distribution pattern and effect on amino acid metabolism.

**Graphical abstract:**

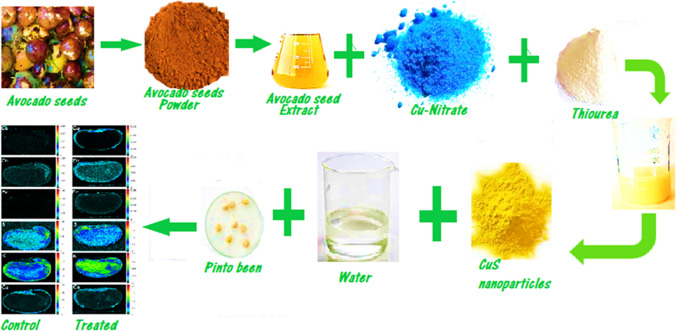

## Introduction

Climate change, an ever-increasing global population demanding high quality food, soaring food costs due to fertilizer shortages, and the adverse impact of the augmented application of chemical and organic fertilizers on the environment and soil fertility collectively support the urgent need to develop sustainable agricultural technologies that can revolutionize crop productivity and quality [1, 2]. To address these broad-based challenges, the development and utilisation of so-called nanotechnology-enabled fertilizers or nanofertilizers to substitute traditional agrochemicals, have presented with significant prospects in research and agriculture due to their unique physico-chemical make-up, nano-size dimensions, and high area to volume ratio facilitating enhanced nutrient use efficiency [3]. A variety of studies on seeds and seedling activity of metal-oxide (e.g. MoO, CeO_2_, CuO, TiO_2_, and ZnO) [4–6] and metal sulphide (e.g. MoS, Ag_2_S, ZnS, FeS, MnS) [4, 7–10] nanoparticles has been done and showed that metal based nanoparticles impact on plant growth vary based on the type, size, shape, concentration and seed/plant species. In contradictory, it has currently been noted that nanoparticle synthesis and test methods also have an influence on the development of the plant [11]. Some nanoparticles have shown no influence at all, a study on molybdenum oxide and sulphide nanoparticles showed that the growth of *Oryza sativa L*. was not improved by 100 ppm concentrations of the nanoparticles [4].

A growing number of studies on uptake and seedling activity of particles are focusing on utilizing metal-based nanoparticles such as copper-sulphur-based nano fertilizers to enhance plant nutrition and growth due to their unique effect on plant metabolic activities, crop resilience to pathogens, and survival of plants under abiotic stress [12–14]. However, reports focused on the effect of Phyto engineered CuS nano fertilizers on plant physiology are lacking. Biogenic metallic nanoparticles developed using Phyto engineering presents as an eco-friendlier, energy efficient, economical, and timeous one-pot synthesis approach towards the large-scale production of highly biocompatible and multifunctional nanoparticles that have been shown to have unique Physico-chemical properties in terms of size, shape, composition, surface functional chemistry, and chemical stability that may have unique benefits to plants [15–20]. Furthermore, although the interaction between different plant phytochemical compounds and nanoparticles plays a critical role in phyto-engineering a product with unique properties, computational studies delineating the interaction between the plant materials used in Phyto engineering and the nanoparticle surface are lacking for metal-based nanofertilizers.

Although nanofertilizers have been applied as foliar or soil nutrition, seed priming with metal-based nanofertilizers presents with added advantages since its application can be performed at one of the most important stages of a plant’s life cycle and under controlled conditions [21–23]. Ultimately, seed nanopriming with green synthesized metal-based nanofertilizers may present as an eco-friendlier approach to applying more bioeffective fertilizers [24]. Seed priming studies with nanofertilizers have shown that nanofertilizers have a multifaceted effect on seed physiology [21] However, most metal or Cu-based nanofertilizer studies have mainly focused on seed physiology in terms of water uptake and germination parameters such as germination time and rate combined with other plant physiological parameters [25]. Essentially, nothing is known about the fundamental interaction of green synthesized CuS-based nanofertilizers with seeds in terms of its effect on amino acid metabolism which may well reflect the plant’s stress response metabolism to the application of a non-traditional fertilizer. Furthermore, nothing is known about the distribution of nanofertilizers in seed morphological regions that may have important implications for seed protection and nutrition.

The aim of this study was to screen the interaction of phytoengineered CuS nanoparticles with Pinto beans (*Phaseolus vulgaris* L.) as bean crops serve as a nutritional security cash crop that has a lower carbon footprint, energy consumption, land and water use [26, 27]. We describe here (1) the molecular dynamics simulation [28–33]; motivated phytoengineering of CuS nanoparticles (2) their distribution inside seeds morphological regions and effect on the seed ionome using external beam proton induced X-ray emission; and (3) their effect on amino acid metabolism.

## Experimental

### Preparation of avocado aqueous extract and nanoparticle

To prepare the aqueous avocado seed extract, the seeds collected from waste were washed with DI water and grated with a blender to obtain the seed powder which was dried overnight in an oven at 50 °C. An aqueous solution of avocado seed extract was prepared as previously described [34]. CuS nanoparticles were prepared by modifying a previously reported method [35]. The dried powder was annealed under vacuum at 400 °C for 2 h to obtain CuS nanoparticles.

### Preparation of imbibing solutions and seed imbibing

The nanoparticle solution was freshly prepared at a concentration of 0.3 mg/ml [36] by dispersing the nanoparticles in DI water using ultrasonic vibration (100 w, 40 kHz) for 30 min. Only deionized water was used for the control treatment. Healthy Pinto bean seeds that represented one cultivar were obtained from Seeds for Africa (Rivergate Industrial, Cape Farms, South Africa) and healthy seeds selected from the same seed lot and used for all the experiments. The seeds were rinsed several times with DI water and immediately soaked for 24 h with continuous aeration in the nanofertilizer solution at a concentration of 0.3 mg/ml and seed weight: solution volume of 1:4 gmL^−1^.

### External beam PIXE analysis

The area on all samples was subsequently measured with a proton beam (spot size = 50 µm) of an initial energy of 3 MeV that decreased to approximately to 2.98 MeV on the target due to energy loss in the exit window of 200 nm Si_3_N_4_ and air gap of 4 mm. All samples were measured with an ion current of 1–3 nA. Typically, about 10.000 points were selected for each map and the dwelling time in particular point was 3–4 s. The energy range of the detected X-rays was set between 1.5 and 30 keV. Emitted X-rays were detected by a Si (Li) detector (resolution, 145 eV at 5.89 keV, 4.3 cm from target, 135° angle to the beam direction). Apart from the air space between the sample and detector, the X-rays passed a pinhole (funny) filter of 50 μm aluminium foil (relative opening, 10%) to balance the intense peaks of light elements and low intensity peaks of metal elements. The transmission function was carefully measured and fitted to an analytical function. The spectra were normalized according to the signal from the chopper that intersected the beam in vacuum. The measured quantity included the RBS signal from the gold foil mounted on chopper wings. The spectral fitting was performed by the Xantho code and elemental concentrations were determined according to the instrumental constants that was determined from the measurement on NIST 620 standard glass. For test measurements, we measured the tomato leaves standard NIST 1573a as an unknown target, whilst for the analysis of bean seeds the analysed elements were assumed to be imbedded in a cellulose matrix.

### Seed amino acid extraction and quantification

The seeds were frozen in liquid nitrogen using a liquid nitrogen pre-chilled pestle and mortar and ground to a fine powder. The method for amino acid extraction and analysis was completed as described in [37]. After imbibition, the dry weight of the samples was recorded. The seeds were subsequently frozen in liquid nitrogen using a liquid nitrogen pre-chilled pestle and mortar and ground to a fine powder after the liquid nitrogen had evaporated. A 0.5 ml solution containing 6 M of HCl with Norleucine (250 ppm) as the internal standard was added to the dried seed powder for protein hydrolysis. AccQ-Tag derivates of amino acids were extracted using an AccQ-Tag Ultra Derivatization Kit according to the manufacturer’s instructions (Waters). A Waters Acquity Ultra Performance Liquid Chromatograph system equipped with a photodiode array detector (260 nm), binary solvent delivery system, and an auto sampler was used to analyze the derivatized amino acids. The sample/standard solution (1 µL) was injected into the mobile phase, which conveyed derivatised amino acids onto a Waters AccQ-Tag Ultra C_18_ column (2.1 × 1000 mm × 1.7 µm) maintained at 60 °C for separation. Amino acids in the samples were identified by co-elution with an amino acid standard H (Pierce) and commercially available individual amino acids (Sigma). Instrument control and data acquisition was performed by MassLynx software which integrates the peaks at the defined retention times and plots calibration curves for each amino acid based on the peak response (peak area/internal standard peak area) against concentration.

### Molecular dynamics simulation

The molecular dynamics simulations were conducted using GROMACS 2019 software [30, 31, 38], employing periodic boundary conditions along with the CHARMM36 force field [32, 39] and the SPC water model [40]. Additionally, the TIP3P model was utilized for the water molecules within the complex [31, 34]. The energy of the systems was optimized using the steepest descent algorithm for all atoms [41]. The NVT ensemble (constant number of particles (N), volume (V), and temperature (T)) coupled to the V-rescale thermal bath at 300 K for 200 ps and the NPT ensemble coupled to the Berendsen pressure bath at 1 atm for 300 ps were used to equilibrate each system. Subsequently, each system was subjected to a 50 ns molecular dynamics simulation with a time step of 1 fs and constant conditions of 1 atm and 300 K. The H-bond lengths were constrained using the LINCS algorithm [42]. Long-range electrostatics were applied using the particle mesh Ewald [43] method. The trajectory data was analyzed using GROMACS utilities, and molecular graphics and visualizations were created using VMD 1.9.3 [44]. CHARMM CGenFF [45] was used to calculate the force fields of CuS and compounds. Six active compounds (4-hydroxybenzoic acid, citric acid, protocatechuic acid, pyrocatechol, quinic acid, and succinic acid) had their electronic structures determined using the Gaussian program, version 09 [46]. The geometry of the molecules was optimized at the B3LYP/6–311++g (d, p) level of theory.

### Statistical analyses

All the analysis were performed in triplicate. For the amino acid data, the data were summarized using summative statistics and an empirical approach used. An unpaired t-test was further used to compare means with significance set at *p* < 0.05. Secondary statistical analysis was performed using a heatmap as previously described [47] and as both a visualization and partitioning tool for the amino acid metabolome data.

## Results and discussion

### Characterization of CuS nanoparticles

**Fig. 1:**
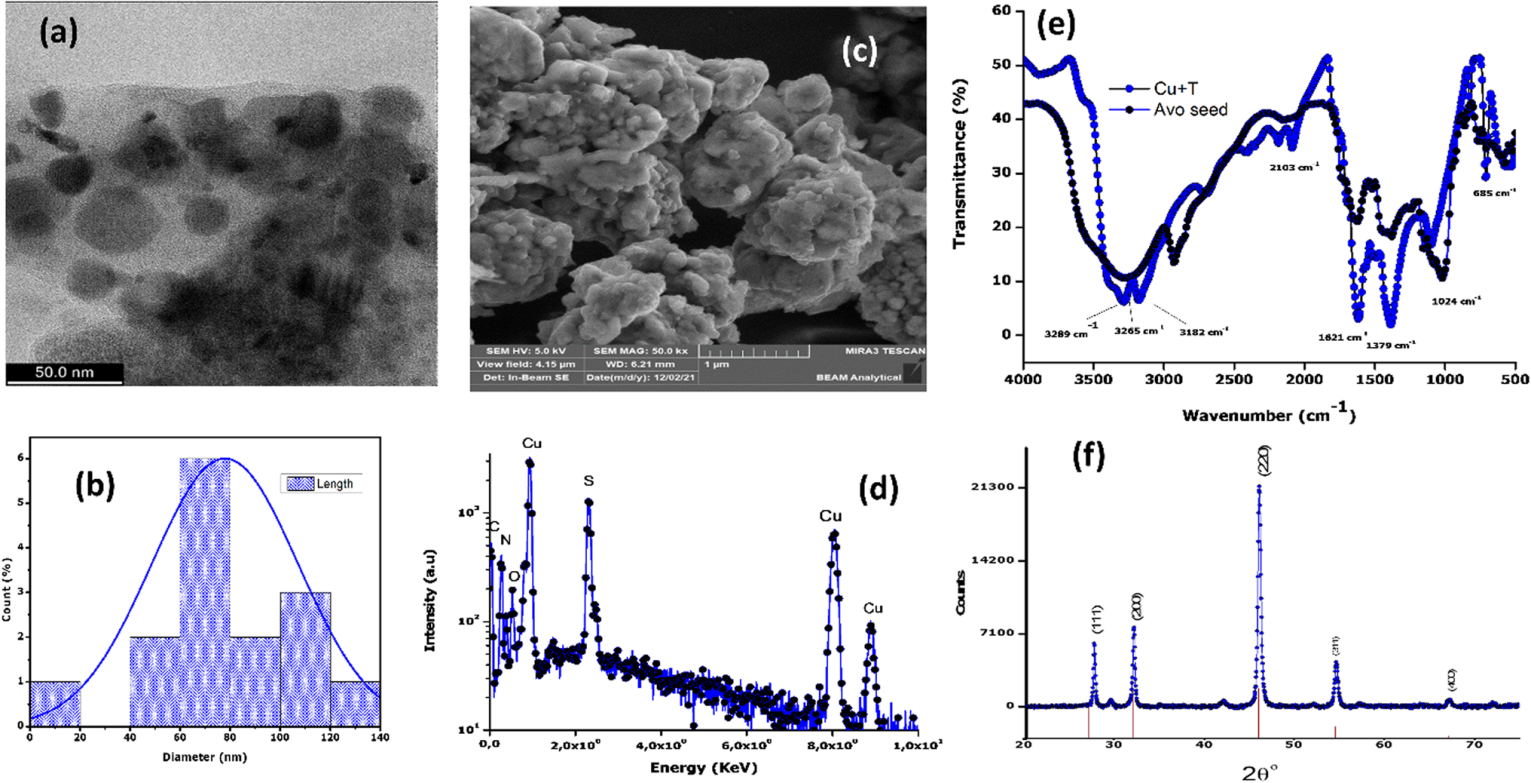
**a** TEM image, **b** size distribution histogram, **c** SEM image **d** EDS spectra, **e** FTIR spectra, and **f** XRD Patterns of CuS nanoparticles

The TEM image (Fig. 1a) at 50 nm scale shows the shape of the particles to be a mixture of spherical and semi spherical with some degree of agglomeration, with an average size of 77.89 nm as shown by the histogram (Fig. 1b). SEM image (Fig. 1c) shows that the synthesised CuS nanoparticles have a rough surface morphology that matches with the previously described CuS nanoparticles [48]. EDX (Fig. 1d) detected carbon, nitrogen and oxygen which could originate from phytocompounds in the avocado seed extract [49], with the presence of Cu and S confirming the formation of the CuS nanoparticles. The presence of phytochemicals such as phenols, alcohols, amines, and carboxylic acid that are known to be present in avocado seeds were further confirmed by comparing the avocado seed powder to the CuS nanoparticle FTIR spectrum (Fig. 1e), both spectra show almost identical vibration peaks [50]. One peak at 694 cm^−1^ from the avocado seed split into two peaks in the CuS and appeared at 769 and 688 cm^−1^ confirming the incorporation of CuS into the chemical structure. XRD in Fig. 1f shows no diffraction peaks associated with impurities, However, only pure digenite (Cu_9_S_5_) phase obtained in reference to JCPDS Cards No. 00-047-1748. The diffraction peaks obtained matched the diffraction peaks obtained in the literature [51]. The Debye–Scherrer equation (D = kλ/βCosθ) was employed to calculate the average size of the Cu_9_S_5_ nanoparticles from the most intense peak to be 70 nm [52, 53].

### External beam PIXE analysis

**Fig. 2:**
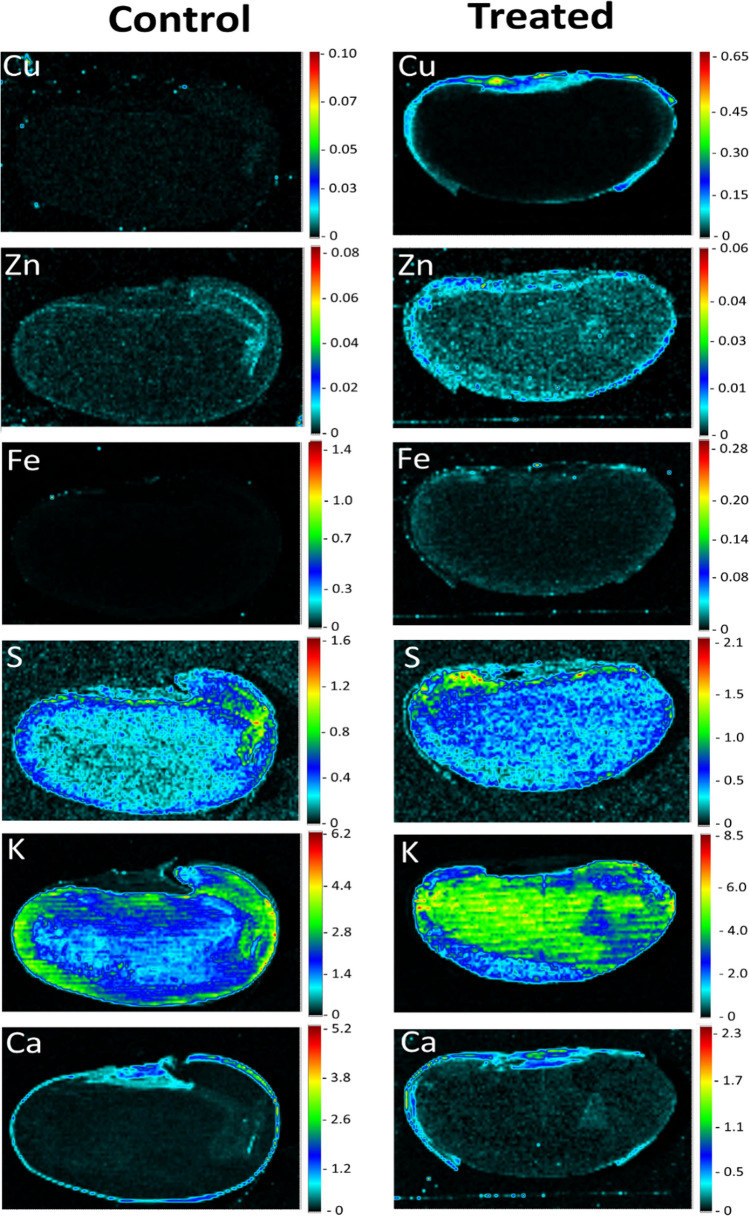
Elemental distribution maps of Cu, Zn, Fe, S, K, and Ca in CuS nanofertilizer treated and control Pinto bean (*Phaseolus vulgaris* L.) seeds treated with water. The intensity scale emphasizes certain elemental distribution features

**Fig. 3:**
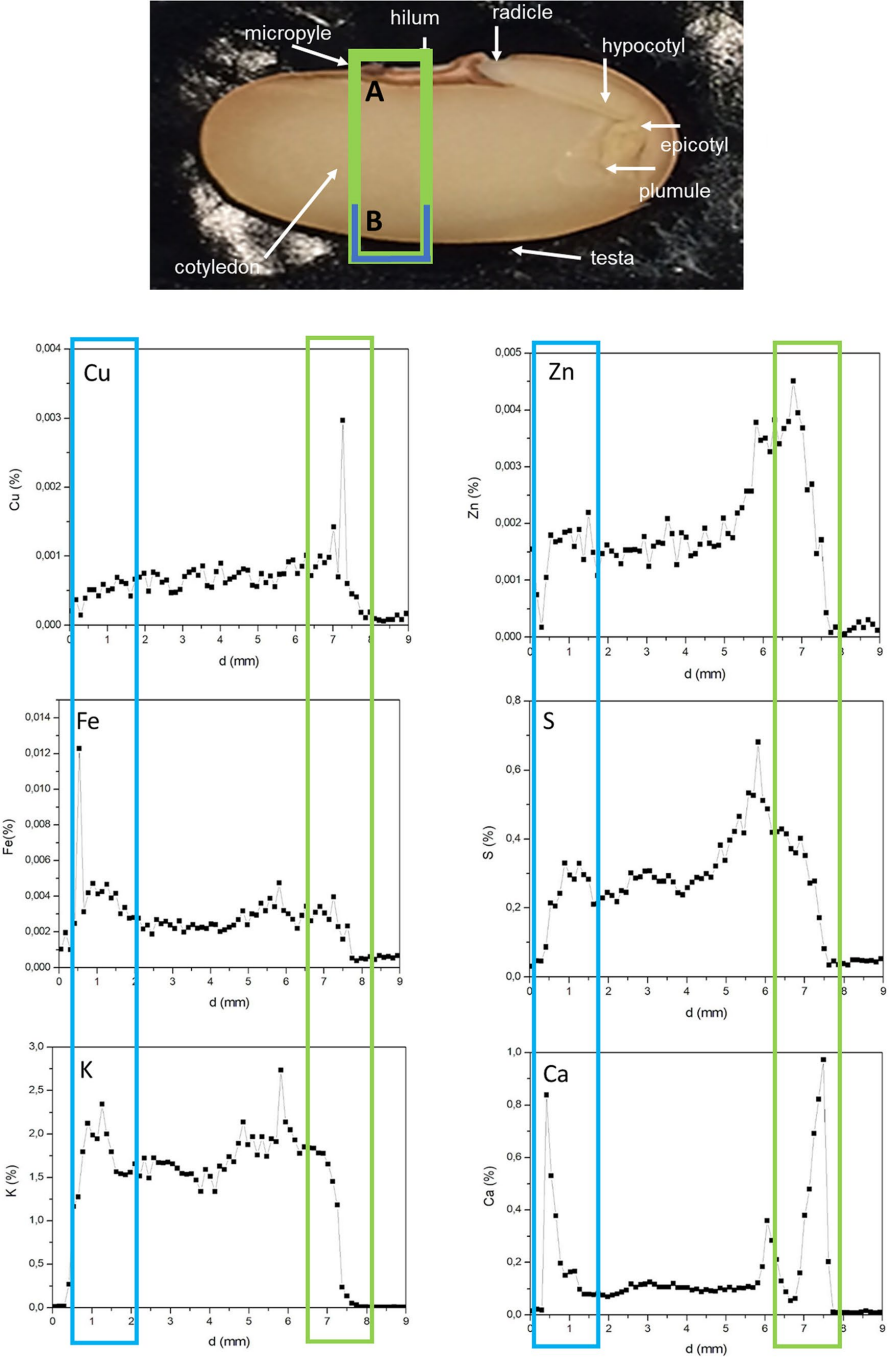

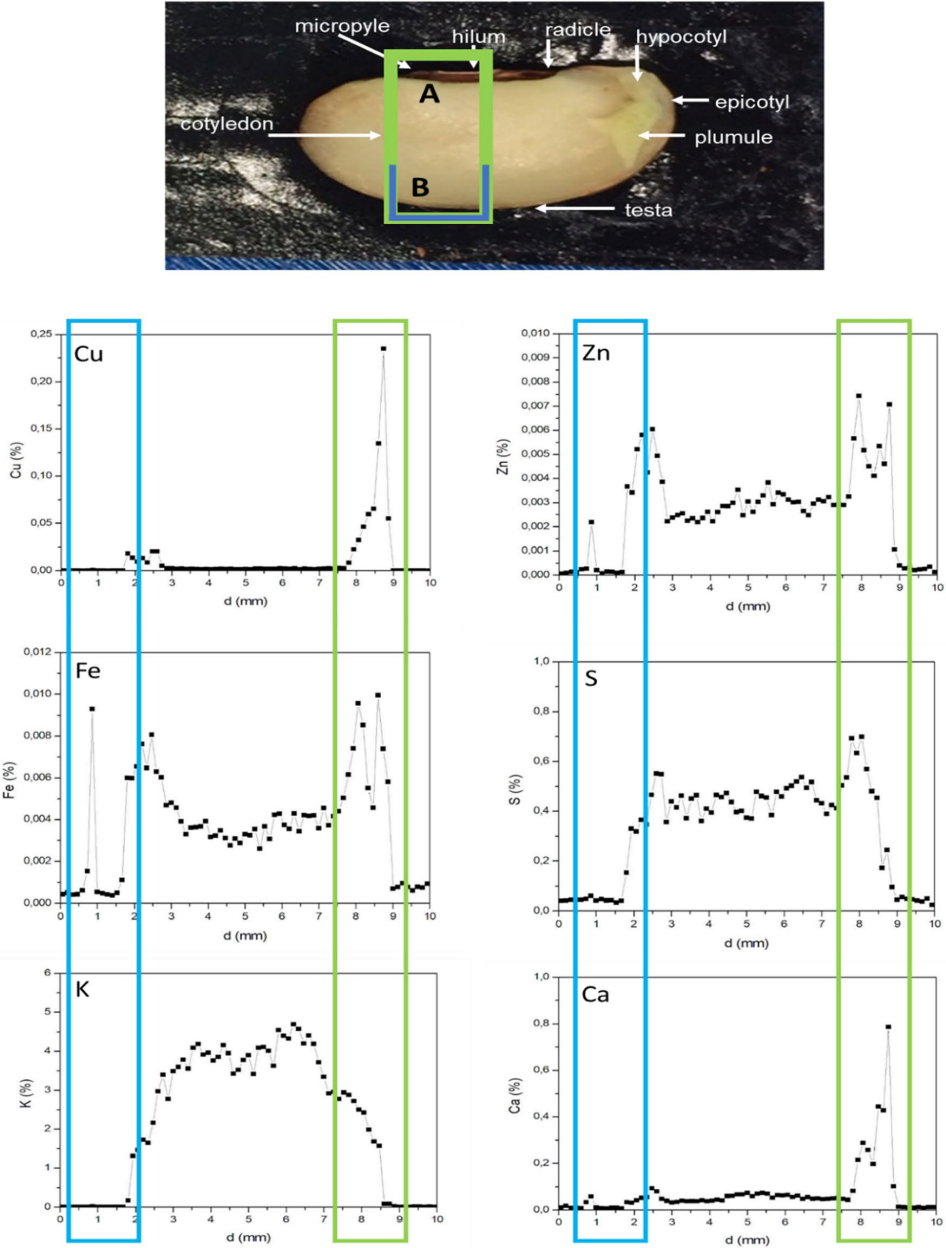
Semi-quantitative (in weight percentage) line scans performed for areas marked in green (**A**) and blue (**B**) linked to different seed morphological regions. The top image presents control Pinto bean (*Phaseolus vulgaris* L.) seeds treated with water, whilst the bottom image represents nanofertilizer treated Pinto bean seeds, The line scans confirm that Cu, Zn, Fe and Ca were mostly accumulated within the seed coat regions, whereas K accumulated within the cotelydon region

Figures 2 and 3 presents the elemental maps of Cu, Zn, Fe, S, K, and Ca distribution intensity obtained from cross-sectioned beans representing nanofertilizer imbibed and control samples imbibed with only deionized water and corresponding semi-quantitative line scan maps across the seed morphological regions, respectively. Some maps, i.e., those of Cu and Fe in the control samples presenting with lower counting statistics and less visually convincing results, were also included since it consistently showed dissimilar distribution patterns between test and control samples. The analysis revealed that the elemental distribution patterns differed between treated and control samples in terms of distribution and intensity and that specific elements tended to accumulate in specific seed morphological regions. For example, in treated samples, Cu was mainly accumulated within the micropyle, hilum, and seed coat regions; Zn, Fe, and Ca were more concentrated in the seed coat region; S showed hotspots in the seed coat close to the hilum and micropyle regions like Cu; and K accumulated within the cotyledon. In control samples, Zn distribution seemed more prominent in the seed coat, S showed some hotspots in the testa and seed coat regions, whilst Ca was mainly located within the seed coat regions. For K, higher intensities were noted within the testa and seed coat regions. Overall, K, Cu, S, and Zn showed notable variation in distribution and intensity patterns between control and treated samples.

The results of the elemental mapping data agree with the study [46] on the localization of chemically synthesized and coated iron oxide nanoparticles in tomato seeds. For beans, previous studies have also shown that, depending on the bean cultivar, Cu, Zn, Fe, and Ca are mainly localized within the seed coat, whereas K tend to accumulate more within the internal seed regions [27]. The distribution patterns observed here may be ascribed to a physiological mechanism whereby bean seeds store specific elements within specific morphological regions [54]. For example, Ca has been shown to have restricted movement between the seed coat and embryo due to the presence of insoluble calcium oxalate crystals in the mature seed coat [27, 55]. Specific elements may also have a biological function linked to a specific seed morphological region [56], whilst seed chemistry may likewise play a significant role as seed coat tannins or other biocompounds such as amino acids may complex metal containing compounds [27, 57]. Ultimately, differences in the intensity and distribution patterns for Cu, Zn, Fe, S, and K between the treatments could be ascribed to the effect of the nanofertilizer on the intricate physiological and metabolic mechanisms governing uptake, distribution, and storage of elements in seeds. More specifically, the distinct distribution patterns for K between test and control samples could be ascribed to the binding of Cu ions to metallothionein proteins in the seed embryonic region [58] which may impact the activity of phytases [59]. The end products of phytate breakdown may in turn affect the activity of K channels which mediates K transport between seed morphological regions [60]. Alternatively, the distribution pattern and dynamics of K movement could be a response to the seed’s multistage hydration dynamics mediated by the nanofertilizer [61], beginning at the micropyle and hilum and filling the voids between the cotyledons and seed coat [62, 63].

Interestingly, the CuS nanofertilizer were mainly accumulated within the seed coat area that supports previous studies showing that nanofertilizers mainly accumulate within this area [64]. More specifically, the nanofertilizer showed hotspots within the hilum and micropyle regions that act as a hygroscopic valve responsible for water uptake*.* On a microlevel nanofertilizers may bind to carrier proteins, ion channels, or membrane transporters [65]. It has also been hypothesized that nanofertilizers may enter plant material via intercellular spaces, pore size enlargement, the induction of new cell wall nanopores, or via endocytosis [66–68]. The creation of nanopores may further upregulate aquaporin production and water uptake [67], hence facilitating the entry of an aqueous nanofertilizer solution into the seed [69, 70]. Alternatively, the nanoparticles forming part of the nanofertilzer may attach to and create nanopores within the spongy like and stacked tissue layers of the hilum containing branched*,* dead tracheid cells with lignified cell walls [71].

Other than the multifaceted chemistry*,* physiology, homeostasis, and metabolism of the plant region exposed to the nanofertilizer, the physico-chemical properties, i.e. size, morphology, chemical composition, surface functionalization, and concentration of the individual nanoparticles that constitute the nanofertilizer may also have a multidimensional effect on nanofertilizer uptake [67, 72]. Here, the functionalization and coating of the nanofertilizer [53] or its low solubility [73] that may serve as a barrier to transport into the seed cotyledon, may further explain restriction of the nanofertilizer to the seed coat area.

Nevertheless, although the interaction between nanofertilizers and the seed coat is generally poorly explored, the presence of the CuS nanofertilizer in the seed coat region presents with significance. Since the seed coat is responsible for protecting the internal seed regions against biotic and abiotic onslaughts [70, 74], accumulation of a Cu-based nanofertilizer in this region may potentially act as a barrier to infective agents such as fungi due to the antimicrobial properties of metal nanoparticles [75]. Furthermore, since the nanofertilizer accumulated in the seed coat, its application may present as versatile carrier platform for not only seed pest control, but also to deliver nutrients or growth factor agents during the early stages of plant development [53]. Interestingly, the seed coat also acts as a channel transmitting cues about the external environment [76]. To illustrate, external nutrients in the form of a nanofertilizer that may eventually pass the seed coat to the internal seed regions to initiate seed development may also trigger downstream macromolecular responses to alter seed metabolism in response to external environment changes [77].

### Amino acid profiling

**Fig. 4:**
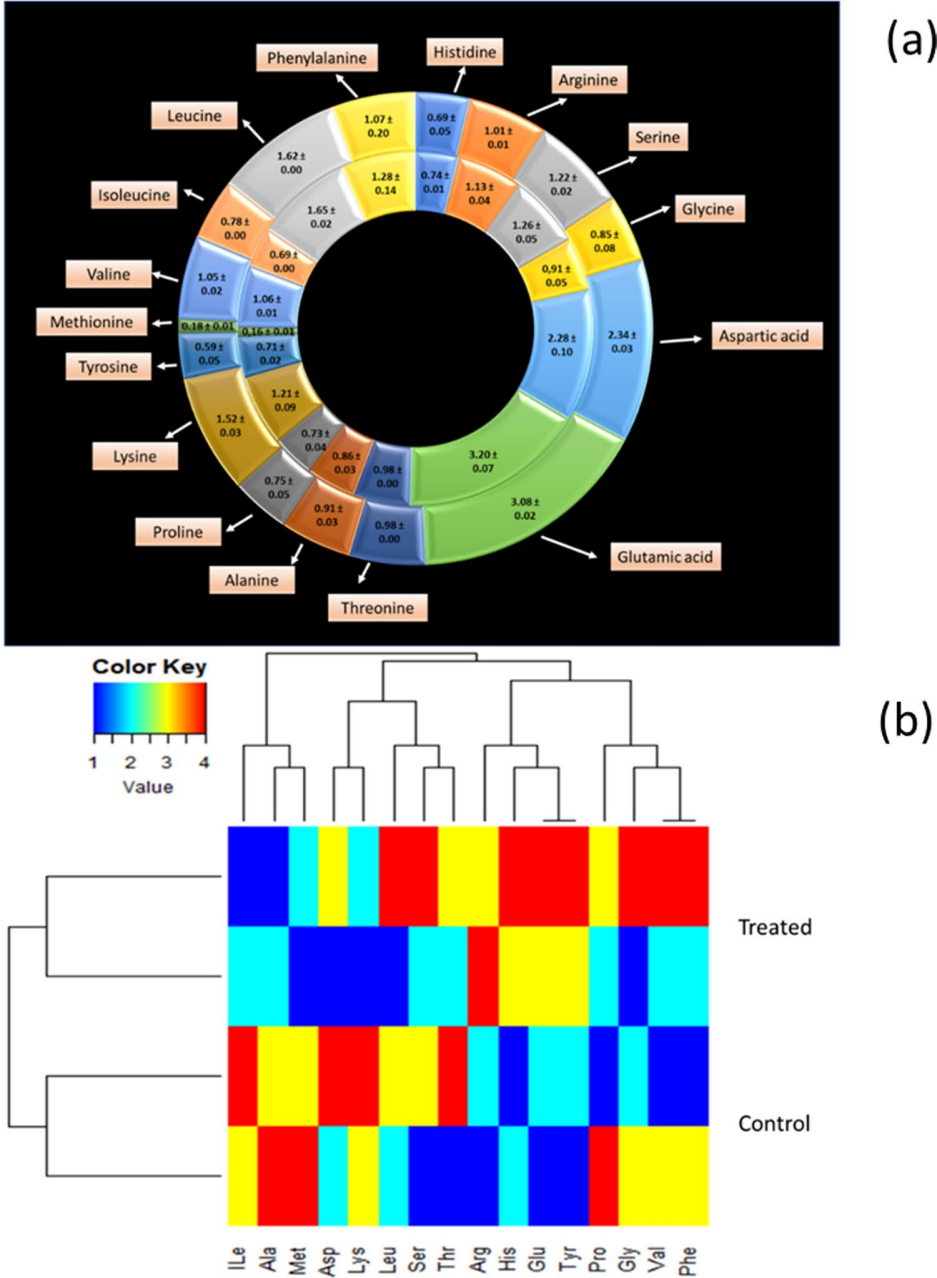
Doughnut graph **a** depicting amino acid levels between CuS nanofertilizer treated Pinto bean seeds (inner doughnut chart) and seeds treated with only water (control, outer doughnut chart). Values in the chart are expressed as mean ± SD % g/100 g dry weight of samples. **b** presents a heatmap generated using complete linkage hierarchical clustering based on Euclidean distances showing the median amino acid abundances (quantified as moderated z-scores) of clusters for amino acid compositions of Pinto bean seeds treated with CuS nanofertilizer and control seeds treated with only water. The relationship is presented by a dendrogram in which rows represent treatment and columns amino acid content, with a specific colour representing the magnitude of abundance (please refer to the text for a full explanation of the heatmap results)

Nanofertilizer treated and control seeds were analyzed for 16 essential and non-essential amino acids. The mean ± SD of amino acid content in treated versus control seeds are presented in Fig. 4a. Descriptive statistical analysis showed that some of the amino acids were slightly upregulated in treated seeds, whilst others were slightly downregulated. Phenylalanine showed the highest increase of 20%, whilst lysine showed the highest decrease of 20% between test and control samples. No significant differences (*p* > 0.05) were noted for amino acid levels between the treatments.

Secondary statistical analysis with hierarchical clustering showed the similarities and differences in the amino acid contents based on individual treatments and the similarity or differences between individual treatments based on amino acid contents. The colour legend of the heatmap (Fig. 4b) indicates a lower average amino acid concentration (blue) or a higher average amino acid concentration (red) in a particular sample. The nanofertilizer treated seeds were differentiated from and showed the highest dissimilarity (as shown by the brightest colours of the colour map) to the control treatment by more extreme z-scores in the negative or below average and positive or above average direction for specific subsets of amino acids compared to the control treatment. Interestingly, isoleucine, alanine, and methionine formed a separate cluster with the rest of the amino acids that formed part of a bigger cluster. This clustering pattern could be linked to the multifunctional interaction of the amino acid groups related to their unique chemistry and physiological roles [78, 79]. Collectively, the heatmap results support and build on the summative statistical analysis.

In sum, it has been shown that metal nanoparticles trigger changes in biochemical reactions by for example downregulating or upregulating metabolites such as amino acids that may affect the plant’s adaptive reaction to changing environmental conditions [80, 81]. Quantitative determination of amino acids is hence important in mapping the metabolome response to environmental cues. Of the amino acids, lysine and phenylalanine showed the most marked response between treatments. Lysine—a nutritionally essential amino acid that at high concentrations may retard germination—was downregulated in test versus control samples [82]. Phenylalanine representing a non-essential amino acid and involved in the defence system of a plant [83, 84] was however upregulated in test versus control samples, although not at a significant level. Furthermore, glutamic acid that plays a significant role in nitrogen metabolism, amino acid and protein synthesis, and the synthesis of phytochelating agents known for binding metals [85, 86] was slightly upregulated together with arginine that also serves as an important nitrogen reserve and precursor for the biosynthesis of polyamines and nitric oxide which regulates nutrient uptake and activates disease and stress tolerance mechanisms in plants [87]. Tyrosine that serves as a precursor of various specialized metabolites such as electron carriers, antioxidants, and defence compounds was also slightly upregulated [88]. Only aspartic acid which serves as a central building block in nitrogen and carbon metabolism and biosynthesis of other amino acids, nucleotides, organic acids, sugars, and other compounds vital for plant growth and stress resistance [89] was slightly downregulated in addition to isoleucine that is known to play a role in plant stress resistance as an osmo-regulation factor [90].

### Molecular dynamics simulation

**Fig. 5:**
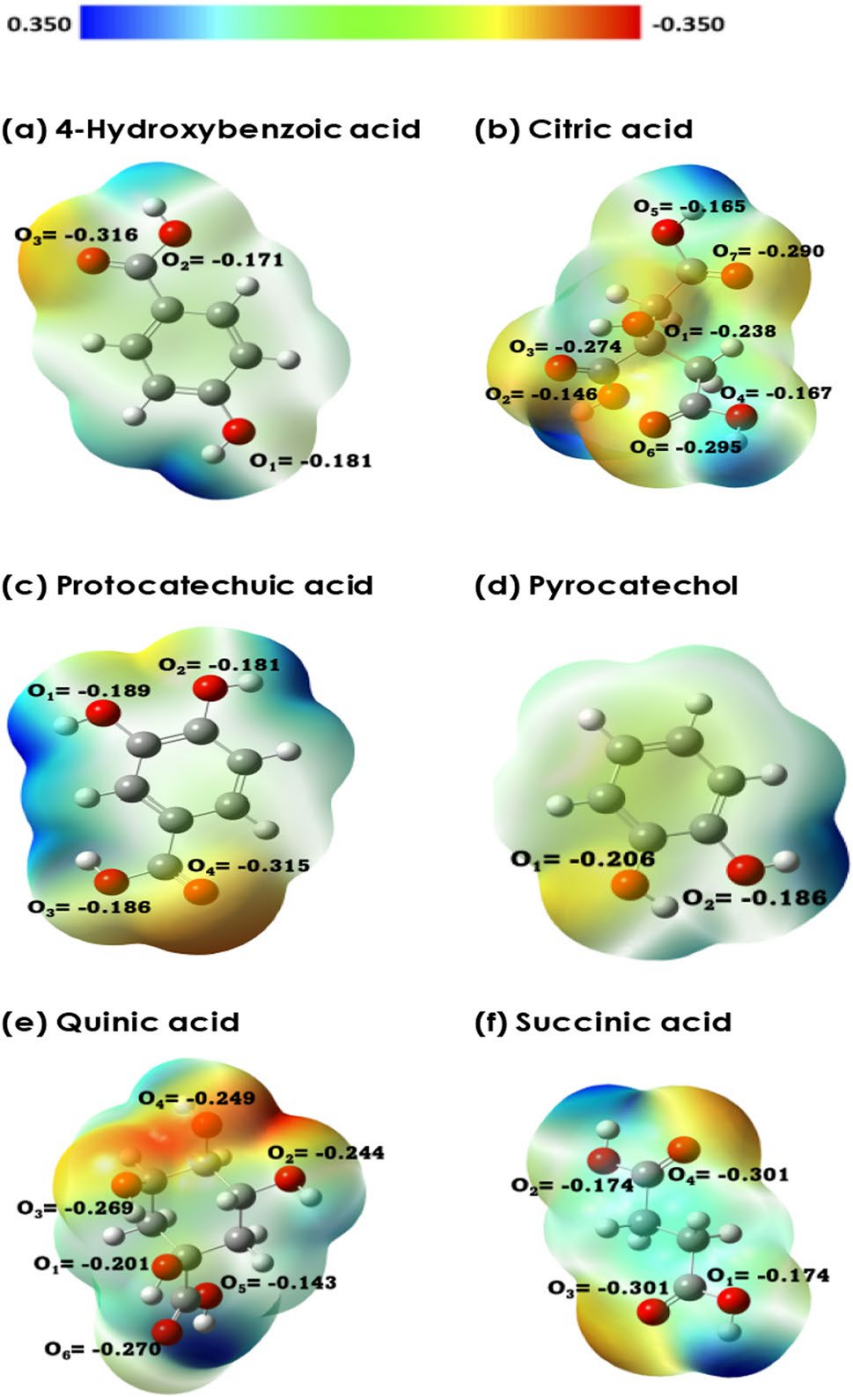
Charge distribution (Hirshfeld point charges) and electrostatic potential map of **a** 4-hydroxybenzoic acid, **b** citric acid, **c** protocatechuic acid, **d** pyrocatechol, **e** quinic acid, and **f** succinic acid. The geometry optimization of active compounds was carried out at the B3LYP/6–311++g(d,p) level of theory

Figure 5 illustrates the charge distribution and electrostatic potential maps of six active compounds, highlighting their active interaction sites with the nanoparticle surface. We derived the Hirshfeld point charges and electrostatic potentials for these compounds' optimized structures through density functional theory, using water as a solvent. The most intense red regions in these maps indicate where the molecules are most likely to interact with the nanoparticle. Specifically, the atoms with the strongest attraction to the CuS surface are O3 in 4-hydroxybenzoic acid, O6 in citric acid, O4 in protocatechuic acid, O1 in pyrocatechol, O3 in quinic acid, and O3/O4 in succinic acid.

**Fig. 6:**
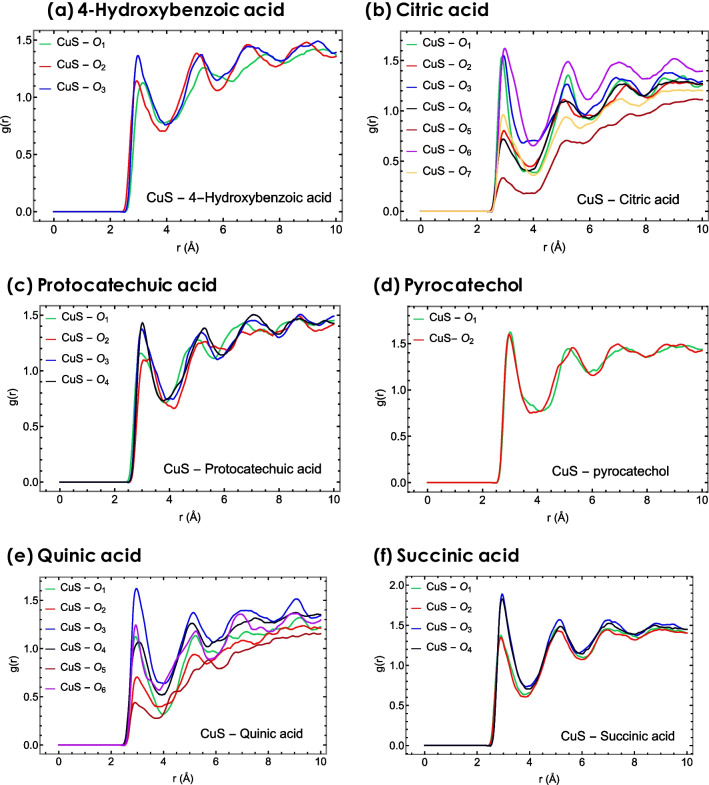
Radial distribution function plots for the active sites of **a** 4-hydroxybenzoic acid, **b** citric acid, **c** protocatechuic acid, **d** pyrocatechol, **e** quinic acid, and **f** succinic acid with respect to the surface of CuS nanoparticle

As depicted in Fig. 6, the g(r) has a maximum peak for the atoms O_3_ in 4-hydroxybenzoic acid, O_6_ in citric acid, O_4_ in protocatechuic acid, O_1_ in pyrocatechol, O_3_ in quinic acid and succinic acid, which exhibit the highest affinity to interact with the nanoparticle compared to other types of oxygen and carbon atoms, as was determined by DFT calculations as well.

LogS is a logarithmic measure of a molecule’s solubility in water, expressed as the base-10 log of its solubility in moles per liter. This metric is a reliable indicator of a compound’s hydrophilicity. Specifically, LogS values above − 1 signify a molecule’s high polarity and affinity for water [91]. Moreover, molecules with LogS values ranging from − 1 to − 5 exhibit a balanced profile of water-solubility and lipophilicity, enabling them to associate with hydrophobic entities [91, 92]. Utilizing Playground v1.5.0 software, the LogS values for various compounds were calculated to assess their solubility in water [93]. The outcomes revealed that 4-hydroxybenzoic acid has the lowest solubility with a LogS value of − 1.07. This is followed by increased solubility in protocatechuic acid (− 0.68), pyrocatechol (− 0.52), and even higher solubility in quinic acid (0.16), citric acid (0.40), and succinic acid (0.77), in ascending order.

**Fig. 7:**
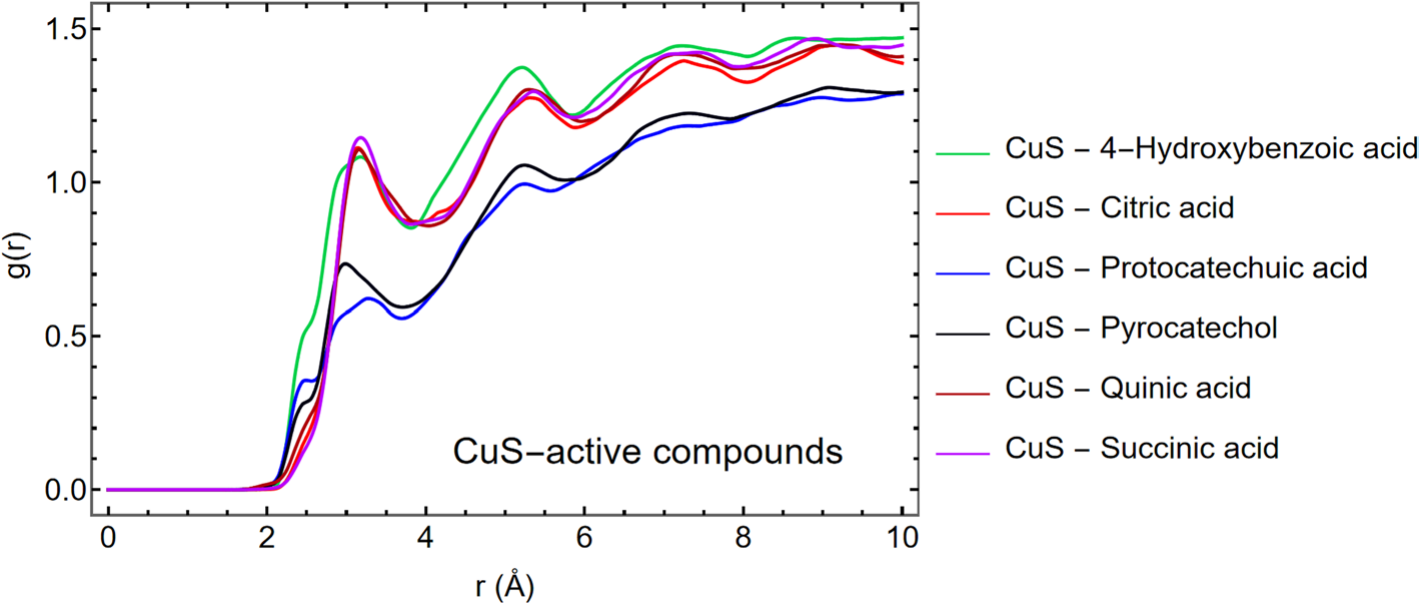
The radial distribution function plots illustrate the spatial distribution of six active compounds in relation to the CuS surface

Figure 7 illustrates that 4-Hydroxybenzoic acid exhibits the most pronounced peak in the radial distribution function (g(r)) at approximately 2.5 Å from the nanoparticle's surface, suggesting a strong affinity for interaction despite its relatively low solubility (logS value). In contrast, succinic acid, which displays the smallest g(r) peak, shows a lower propensity to interact with the nanoparticle surface, correlating with its high logS value, indicative of greater solubility. Additionally, when analyzing the interaction energies, which combine Van der Waals and electrostatic potential energies, a trend emerges: the interaction energy is higher near the surface of CuS for the 4-Hydroxybenzoic acid than other compounds in an aqueous environment. The data from interaction energies and the radial distribution function (RDF) graphs, as presented in Fig. 6, corroborate the logS values’ implications. They collectively suggest that succinic acid is markedly more hydrophilic compared to other active compounds, thereby preferring interaction with water molecules over the nanoparticle surface.

## Conclusions

To promote our understanding for the rational use of nanotechnology for seed treatment in agriculture, this study presents the first report applying computational modelling supported Phyto engineered CuS nanoparticles as a seed imbibing agent to study its effect on the distribution of elements across different seed morphological regions combined with its effect on the amino acid metabolome using both benchtop and ion beam techniques. Molecular dynamics simulations showed that specific phytocompounds had a higher affinity for interacting with the nanoparticle's surface than other active compounds. The nanofertilizer containing nanoparticles of ca. 77 nm were retained within the seed coat region which generally serves as a barrier for seed protection, nutrient reserve, and response trigger to a changing environment [80]. Although the reasons supporting the preferential and more intense elemental accumulation patterns triggered by the nanofertilizer for some elements are unclear, the intricate crosstalk between the chemico-physiology of the bean regions with the unique Physico-chemical properties of the nanofertilizer as characterized by physico-chemical analyses and computational modelling may play a significant role. The unique changes in the ionome in terms of its distribution between treated and control seeds may have triggered a secondary metabolite response in the form of amino acid level changes, with the slight change observed in amino acid levels not sufficient to indicate a strong plant defence mechanism. Regardless, the fact that the nanofertilizer accumulated within the seed coat regions may have significant implications for the use of this nanofertilizer as a versatile carrier technology to deliver nutrients or antimicrobials [72]. In future, laboratory studies as here described are firstly warranted before applied studies in the field can be executed to screen the growth physiology and performance of seeds treated with nanofertilizers as affected by a host of soil Physico-chemistry and environmental factors. For example, answering fundamental questions is first and foremost important, such as the distribution of the nanofertilizer within seed morphological regions that may also affect for example its seed antimicrobial potential, whilst activation or changes in amino acid metabolism by the nanofertilizer may further serve to improve the resilience of the seed to abiotic and biotic stress.

## Data Availability

The data supporting the findings reported is available upon reasonable request from the corresponding author.
